# A retrospective seroepidemiologic survey of *Chlamydia pneumoniae* infection in patients in Beijing between 2008 and 2017

**DOI:** 10.1371/journal.pone.0206995

**Published:** 2018-11-07

**Authors:** Jingtao Cui, Wenjuan Yan, Hongjie Xie, Shaoxia Xu, Qiaofeng Wang, Weihong Zhang, Anping Ni

**Affiliations:** Department of Clinical Laboratories, Peking Union Medical College Hospital, Chinese Academy of Medical Sciences and Peking Union Medical College, Beijing, China; Midwestern University, UNITED STATES

## Abstract

**Background:**

*Chlamydia pneumoniae (C*. *pneumoniae)* is an obligate intracellular bacterium and a human pathogen that causes respiratory infectious diseases. More than 50% of the adult population worldwide was once infected with *C*. *pneumoniae*, but investigations into this topic are insufficient in mainland China.

**Methods:**

Anti-*C*. *pneumoniae* IgG and IgM antibodies were detected using micro-immunofluorescence test in serum samples of patients visiting Peking Union Medical College Hospital between 2008 and 2017 for routine medical purposes, and the aim of this retrospective study was to analyze the test results.

**Results:**

Among 12,050 serum specimens tested for anti-*C*. *pneumoniae* IgG and IgM antibodies, the overall prevalence of anti-*C*. *pneumoniae* IgG antibodies was 86.6%, 87.2% for men and 86.0% for women. Adult men (>20 years) were found to have a significantly higher prevalence of anti-*C*. *pneumoniae* IgG than women (*χ*^*2*^ = 30.32, *P* = 0.000). 3 to 5 years old patients were observed to have the lowest prevalence of anti-*C*. *pneumoniae* IgG, 42.8%, then increased with age, reaching the highest level of 98.6% in patients over 70 years of age. In the 10,434 specimens with *C*. *pneumoniae* IgG antibodies, the total geometric mean titer (GMT) for *C*. *pneumoniae* IgG was 45.71. Although GMTs were found to be significantly higher among all men than among all women (*t* = 5.916, *P* = 0.000), sex difference actually began in patients over 40 years of age and increased in the elderly. In the total 12,050 specimens, 1.2% had anti-*C*. *pneumoniae* IgM, 3.3% had anti-*C*. *pneumoniae* IgG with titers equal to or greater than 1:512; 0.39% had ≥4-fold increasing titers of antibodies in acute and convalescent phase paired samples, and 4.4% were finally confirmed to have acute antibodies against *C*. *pneumoniae*. 6 to 10 years old patients were found to have the highest rate of both IgM antibodies (3.9%) and acute antibodies (6.2%) against *C*. *pneumoniae*. Acute antibodies against *C*. *pneumoniae* were found to be more frequent in patients with acute exacerbation of chronic obstructive pulmonary disease (AECOPD, 14.0%, *χ*^*2*^ = 20.43, *P* = 0.000), patients with pneumonia (7.8%, *χ*^*2*^ = 51.87, *P* = 0.000) and patients with acute respiratory tract infection (12.3%, *χ*^*2*^ = 60.91, *P* = 0.000) than among all patients (4.4%). Both anti-*C*. *pneumoniae* IgG and IgM antibodies should be tested for acute antibodies against *C*. *pneumoniae* as testing for either alone will underestimate by a maximum of two-thirds the incidence of acute antibodies against *C*. *pneumoniae*.

**Conclusions:**

More than 86% of Chinese patients on an average were once infected with *C*. *pneumoniae*. Adult men had both a higher prevalence and higher levels of antibodies than women. 6 to10 year old patients were found to have the most frequent acute infection of *C*. *pneumoniae*. *C*. *pneumoniae* is associated with AECOPD, pneumonia and acute respiratory tract infection. Anti-*C*. *pneumoniae* IgG and IgM should be tested simultaneously to avoid underestimation of acute antibodies against *C*. *pneumoniae*.

## Introduction

The members of the Chlamydiaceae family are obligate intracellular bacteria, with only a single genus, Chlamydia, currently comprising 11 species [1]. Only 3 of these species are generally involved in human infectious diseases (*Chlamydia trachomatis*, *Chlamydia pneumoniae* and *Chlamydia psittaci)*. *Chlamydia penumoniae* (*C*. *pneumoniae*) was recognized as a new species of Chlamydia in 1989 [2], and it is responsible for human respiratory infectious diseases, such as pharyngitis, bronchitis and pneumonia [2, 3]. More than 50% of the adult populations worldwide were once infected with *C*. *pneumoniae* [2, 3]. *C*. *pneumoniae* was identified as the causative pathogen in 10–15% of adult and pediatric patients with community-acquired pneumonia [2–5]. This bacterium may also play a role in chronic infection in patients with chronic obstructive pulmonary diseases (COPD), including in exacerbations of the disease [6].

In 1996, we conducted a seroepidemiologic study of *C*. *pneumoniae* in small sample of normal population in Fujiang Province, South of China, in which 61.5% of individuals were found to have IgG antibodies against *C*. *pneumoniae* [7]. More than 20 years later, and we tested more than 12,000 specimens from patients in Beijing during the last 10 consecutive years, from 2008 to 2017, for anti-*C*. *pneumoniae* specific IgG and IgM antibodies using micro-immunofluorescence (MIF) testing, the standard method for serology of *C*. *pneumoniae* [3, 8]. Analysis of these test results is worthwhile as there are not sufficient studies on the topic in the mainland China, and we hope to provide some epidemiological data on *C*. *pneumoniae* infection in Beijing, such as prevalence and levels of anti-*C*. *pneumoniae* antibodies in patients with different ages, sexes, and diseases, as well during different seasons, and years, and to explore the clinical meaning of any differences.

## Subjects and methods

### Ethics statement

The study was approved by the Ethics Committee (reference no. S-K362) of Peking Union Medical College Hospital (PUMCH), and the requirement for informed consent was waived, as the blood samples were collected and tested for routine medical service. The information regarding the patients’ identity had been deleted before the analysis.

### Study samples

Between May 2008 and December 2017, 12,050 serum samples were collected from outpatients and hospitalized patients in Beijing and tested at the PUMC Hospital.

Inclusion criteria were samples from patients who: were Chinese, had demographic information and had laboratory test results of anti-*C*. *pneumoniae* antibodies.

Exclusion criteria were samples from patients who: were foreigners (as the study was designed only for Chinese patients), had no demographic information or had no laboratory test results.

Cases of more than one serum samples for the same diagnosis within 3 months were considered as repeated samples, and only first sample was used, with the rests excluded, unless they were acute and convalescent phase paired samples with ≥4-fold increasing titers of the antibodies.

### Laboratory testing

MIF testing was used to detect specific IgG and IgM antibodies against *C*. *pneumoniae* [9, 10]. For details of the testing procedure, please refer to our previous report [7]. Briefly, the antigens were formalized elementary bodies (EBs) of *C*. *pneumoniae* CWL-029 that were grown in Buffalo Green Monkey Kidney (BGMK) cells and purified by step density gradient centrifugation [11]. EBs were applied to slides and fixed with acetone. Twofold serial serum dilutions starting from 1:16 for both IgG and IgM were incubated at 37°C for 30 min for the IgG testing and 90 min for the IgM testing, washed in PBS, rinsed in distilled water, dried and stained with FITC-labelled rabbit-anti human IgG and goat-anti human IgM antibodies (F4512 and F5384, Sigma-Aldrich Corp., St. Louis, MO, USA). The slides were secondly incubated for 30 min for both IgG and IgM testing, washed as described above, and read under a fluorescence microscope (Olympus BX51, Olympus Corp., Tokyo, Japan). Sera with positives for IgM antibodies were absorbed with rheumatoid factor absorption reagent (Eurosorb, Euroimmun Medical Laboratory Diagnostics AG, Lubeck, Germany) to exclude false positive reactions.

Regarding criteria for *C*. *pneumoniae* infection: An IgG antibody titer ≥ 1:512 and/or IgM antibody titer ≥ 1:16 and/or acute and convalescent paired samples with ≥4-fold increasing titers of the antibodies were considered evidence of acute infection and any antibodies meeting the criteria above were defined as acute antibodies; an IgG titer ≥1:16 but < 1:512 was considered evidence of previous infection [2, 3, 8]. Please refer to S1 File in the support information (anti-*C*. *pneumoniae*.xlsx) for the details.

### Statistical analysis

MIF results for anti-*C*. *pneumoniae* IgG and IgM antibodies were stored in our laboratory information system (LIS) after completion of tests and clinical reports. The data were transformed to Microsoft Excel 2007, where they were sorted and preliminarily calculated. Statistical analysis was performed with IBM SPSS software (Version 21.0, IBM Corp., Armonk, NY, USA). *P*-values < 0.05 were considered statistically significant. Pearson’s chi-square test and independent samples *t*−test were used to compare the prevalence and the geometric mean titers (GMT) of anti-*C*. *pneumoniae* antibodies according to patients’ age, sex and diseases.

## Results

### Prevalence of anti-*C*. *pneumoniae* IgG antibodies

There were a total of 12,050 serum specimens from May 2008 to December 2017 that were tested for anti-*C*. *pneumoniae* IgG and IgM antibodies with MIF. The patients’ ages ranged from 1 day after birth to 107 years old (mean age, 43.0 ± 24.5 years; median age, 44 years). There were 6261 (52.0%) specimens from male patients (1day to 107 years) and 5789 (48.0%) specimens from female patients (2 days to 104 years), the mean ages for men and women were 43.7 ± 25.4 and 42.3 ± 23.4 years, respectively.

The overall prevalence of anti-*C*. *pneumoniae* IgG antibodies was 86.6% (10,434/12,050), with a prevalence of 87.2% (5458/6261) for male patients and 86.0% (4976/5789) for female patients. The age- and gender- related prevalence of anti-*C*. *pneumoniae* IgG antibodies in male and female patients are listed in Table 1. The prevalence of anti-*C*. *pneumoniae* IgG antibodies was 63.5% (912/1436) in male patients and 66.2% (811/1225) in female patients who were 20 years of age or less and no sex difference (*χ*^*2*^ = 1.986, *P* = 0.159) was found. However, the sex difference was found to be significant between male and female patients older than 20 years of age (94.2% vs. 91.3%, *χ*^*2*^ = 30.32, *P* = 0.000).

**Table 1:** Age- and sex- related prevalence of anti-*C*. *pneumoniae* IgG antibodies in patients visiting the PUMC Hospital, Beijing, 2008–2017.

	Male *C*. *pneumoniae* IgG Pos no. (%)	Female *C*. *pneumoniae* IgG Pos no. (%)	Total *C*. *pneumoniae* IgG Pos no. (%)	*χ*^*2*^ value	*P* value
0–6m	194(60.2)	91(62.3)	285(60.9)	**1.986**	0.159*
7m–2y	90(60.0)	41(53.9)	131(58.0)		
3–5y	72(41.4)	50(45.0)	122(42.8)		
6–10y	108(56.5)	150(61.0)	258(59.0)		
11–20y	448(74.8)	479(74.1)	927(74.5)		
21–30y	538(84.1)	621(79.8)	1159(84.7)	**30.23**	0.000**
31–40y	570(88.1)	692(87.0)	1262(87.5)		
41–50y	641(94.7)	659(92.9)	1300(93.8)		
51–60y	891(96.2)	735(94.0)	1626(95.2)		
61–70y	883(98.2)	713(96.1)	1596(97.3)		
71y–	1023(98.7)	745(98.3)	1768(98.6)		
Total	5458(87.2)	4976(86.0)	10434(86.6)	**3.741**	0.053***

m, months; y, years

* Comparison of prevalence of anti-*C*. *pneumoniae* IgG antibodies between male and female patients equal to or less than 20 years old.

** Comparison of prevalence of anti-*C*. *pneumoniae* IgG antibodies between male and female patients more than 20 years old.

*** Comparison of prevalence of anti-*C*. *pneumoniae* IgG antibodies between all male and all female patients.

### Geometric mean titers (GMT) of anti-*C*. *pneumoniae* IgG antibodies

Among the 10,434 serum specimens with *C*. *pneumoniae* IgG antibodies, the total geometric mean titers (GMT) of *C*. *pneumoniae* IgG was 45.71, and the GMT in male patients was significantly higher than that in female patients (48.23 vs. 43.10, *t* value = 5.916, *P* = 0.000). To identify the demographic location of the exact difference, the age- and sex- related GMTs are presented in Table 2. GMTs of *C*. *pneumoniae* IgG antibodies in young female patients were slightly higher than those in young male patients who were equal to or less than 20 years old; however, GMTs in men exceeded those in women who were older than 40 years of age, and the largest gap was found between men and women over 70 years old.

**Table 2:** Age- and sex- related GMT of *C*. *pneumoniae* IgG antibodies in patients visiting the PUMC Hospital, Beijing, 2008–2017.

	Male GMT	Female GMT	Total GMT	*t* value	*P* value
0–2y	27.04	30.68	28.15		0.1267
3–10y	31.39	37.92	34.67		0.0781
11–20y	43.34	48.62	45.99		0.1070
21–30y	41.14	40.00	40.53		0.6077
31–40y	40.66	40.45	40.55		0.9239
41–50y	**46.62**	**43.69**	44.87	**2.072**	0.0385*
51–60y	**48.97**	**44.30**	46.80	**2.140**	**0.0325**
61–70y	**59.78**	**46.30**	53.33	**5.139**	**0.0000**
71y–	**64.13**	**46.26**	55.88	**7.198**	**0.0000**
Total	**48.23**	**43.10**	45.71	**5.916**	**0.0000**

y, years

*Comparison of GMT between male patients and female patients aged 41–50 years old, same patterns used for other comparisons in Table 2.

### Prevalence of acute infection of *C*. *pneumoniae*

In the total 12,050 specimens, 141 (1.2%) were found to have anti-*C*. *pneumoniae* IgM antibodies; The age- and sex- related prevalence of anti-*C*. *pneumoniae* IgM antibodies in male and female patients are listed in Table 3. The highest prevalence of anti-*C*. *pneumoniae* IgM antibodies was found among 6 to 10 years old patients for both males and females. 402 (3.3%) were detected to have anti-*C*. *pneumoniae* IgG with titers equal to or greater than 1:512 (Table 4). Moreover, 47 (0.39%) patients were found to have acute and convalescent phase paired samples with ≥ 4-fold increasing titers of antibodies.

**Table 3:** Age- and sex-related prevalence of anti-*C*. *pneumoniae* IgM antibodies in patients visiting the PUMC Hospital, Beijing, 2008–2017.

	Male *C*. *pneumoniae* IgM Pos no. (%)	Female *C*. *pneumoniae* IgM Pos no. (%)	Total *C*. *pneumoniae* IgM Pos no. (%)
0–6m	0(0)	1(0.68)	1(0.21)
7m–2y	1(0.67)	0(0)	1(0.44)
3–5y	1(0.57)	1(0.90)	2(0.70)
6–10y	5(2.6)	12(4.9)	17(3.9)
11–20y	14(2.3)	22(3.4)	36(2.9)
21–30y	12(1.9)	16(2.1)	28(2.0)
31–40y	10(1.5)	12(1.5)	22(1.5)
41–50y	5(0.74)	5(0.71)	10(0.72)
51–60y	4(0.43)	4(0.51)	8(0.47)
61–70y	4(0.44)	4(0.54)	8(0.49)
71y–	3(0.29)	5(0.66)	8(0.45)
Total	59(0.94)	82(1.4)	141(1.2)

m, months; y, years

**Table 4:** Age- and sex-related prevalence of anti-*C*. *pneumoniae* IgG antibodies with titers equal to or greater than 1:512 in patients visiting the PUMC Hospital, Beijing, 2008–2017.

	Male *C*. *pneumoniae* IgG ≥1:512 Pos no. (%)	Female *C*. *pneumoniae* IgG ≥1:512 Pos no. (%)	Total *C*. *pneumoniae* IgG ≥1:512 Pos no. (%)
0–6m	1(0.31)	1(0.68)	2(0.43)
7m–2y	1(0.67)	0(0)	1(0.44)
3–5y	1(0.57)	0(0)	1(0.35)
6–10y	7(3.7)	8(3.3)	15(3.4)
11–20y	24(4.0)	23(3.6)	47(3.7)
21–30y	14(2.2)	15(1.9)	29(2.0)
31–40y	19(2.9)	24(3.0)	43(3.0)
41–50y	29(4.3)	20(2.8)	49(3.5)
51–60y	27(2.9)	22(2.8)	49(2.9)
61–70y	53(5.9)	33(4.4)	86(5.2)
71y–	58(5.6)	22(2.9)	80(4.5)
Total	234(3.7)	168(2.9)	402(3.3)

m, months; y, years

According the criteria for *C*. *pneumoniae* infection described above, 529 (4.4%) of the total 12,050 specimens met the criteria for acute infection of *C*. *pneumonia*e (Table 5). Among these, 291 (4.6%) were male patients and 238 (4.1%) were female patients, with the only significant sex difference found between men and women over 71 years old (6.6% vs. 4.0%, *χ*^*2*^ = 5.262, *P* = 0.022).

**Table 5:** Age- and sex-related prevalence of acute antibodies against *C*. *pneumoniae* in patients visiting the PUMC Hospital, Beijing, 2008–2017.

	Male Acute antibodies Pos no. (%)	Female Acute antibodies Pos no. (%)	Total Acute antibodies Pos no. (%)
0–6m	2(0.62)	1(0.68)	3(0.64)
7m–2y	1(0.67)	1(1.3)	2(0.88)
3–5y	3(1.7)	2(1.8)	5(1.75)
6–10y	12(6.3)	15(6.1)	27(6.2)
11–20y	31(5.2)	43(6.7)	74(5.9)
21–30y	22(3.4)	27(3.5)	49(3.5)
31–40y	25(3.9)	29(3.6)	54(3.7)
41–50y	32(4.7)	24(3.4)	56(4.0)
51–60y	36(3.9)	29(3.7)	65(3.8)
61–70y	59(6.6)	37(5.0)	96(5.9)
71y–	**68(6.6)**	**30(4.0)**	98(5.5)
Total	291(4.6)	238(4.1)	529(4.4)

m, months; y, years

### Yearly and season− related prevalence of acute infection of *C*. *pneumoniae*

Year to year variations on prevalence of acute infection of *C*. *pneumoniae* are shown in Table 6. The lowest prevalence of acute *C*. *pneumoniae* infection was 1.4−2.4% in 2008−2009 and the highest was 6.8% in 2017. The former was statistically lower (*χ*^*2*^ = 8.84, *P* = 0.003) and the latter was statistically higher (*χ*^*2*^ = 18.67, *P* = 0.000) when compared with the average prevalence (4.4%, 529/12,050) of the total 10−year period.

**Table 6:** Year- related prevalence of acute *C*. *pneumoniae* infection in patients visiting the PUMC Hospital, Beijing, 2008–2017.

	*C*. *pneumoniae* IgG Titers ≥1:512 Pos no. (%)	*C*. *pneumoniae* IgM Titers ≥1:16 Pos no. (%)	*C*. *pneumoniae* Acute antibodies Pos no. (%)	*χ*^*2*^ value	*P* value
2008–	6(0.96)	1(0.16)	9(1.44)		
2009–	18(1.68)	10(0.94)	**26(2.43)**	**8.84**	0.003*
2010–	24(3.3)	12(1.66)	30(4.1)		
2011–	17(2.1)	9(1.1)	**24(2.9)**	**4.19**	**0.041**
2012–	48(4.0)	16(1.33)	61(5.1)		
2013–	51(3.8)	21(1.56)	66(4.9)		
2014–	56(4.0)	13(0.93)	69(5.2)		
2015–	41(2.7)	13(0.87)	59(3.9)		
2016–	44(2.7)	18(1.1)	67(4.2)		
2017–	97(5.6)	28(1.6)	**118(6.8)**	**18.67**	**0.000**
Total	402(3.3)	141(1.2)	**529(4.4)**		

* Comparison of prevalence of acute *C*. *pneumoniae* infection between patients from year 2009 (2.43%) and all patients on average over 10 year period (4.4%, 529/12,050) of study, same patterns used for other comparisons in Table 6.

The four seasons were defined as spring (March to May), summer (June to August), autumn (September to November) and winter (December to February). There were 9 seasons of spring, 10 seasons of summer, autumn and winter. Season related prevalence of acute infection of *C*. *pneumoniae* is shown in Table 7. Variations in the prevalence of acute infection of *C*. *pneumoniae* in the 4 seasons were not found to be significantly different (*χ*^*2*^ = 4.20, df = 3, *P* = 0.240).

**Table 7:** Seasons- related prevalence of acute *C*. *pneumoniae* infection in patients visiting the PUMC Hospital, Beijing, 2008–2017.

	*C*. *pneumoniae* IgG Titers ≥1:512 Pos no. (%)	*C*. *pneumoniae* IgM Titers ≥1:16 Pos no. (%)	*C*. *pneumoniae* Acute antibodies Pos no. (%)
Spring–	100(3.4)	43(1.44)	130(4.4)
Summer–	119(3.8)	36(1.16)	150(4.8)
Autumn–	96(3.2)	29(0.97)	137(4.6)
Winter–	87(2.9)	33(1.1)	112(3.8)
Total	402(3.3)	141(1.2)	529(4.4)

### Incidence of acute infection of *C*. *pneumoniae* in different patients

Among the 107 patients with acute exacerbation of chronic obstructive pulmonary disease (AECOPD), 14% (15/107) met the criteria for acute infection of *C*. *pneumoniae*, which was significantly higher (*χ*^*2*^ = 20.43, *P* = 0.000) than 4.4% in the total 12,050 patients. Furthermore, 2585 specimens were collected from the patients with pneumonia, 462 from patients with acute respiratory tract infection and 3208 from patients with fever of unknown origin (FUO). All comparisons of the prevalence of acute *C*. *pneumoniae* infection between these different patient groups and the total patient population are shown in Table 8.

**Table 8:** Incidence of acute infection of *C*. *pneumoniae* in different patients visiting the PUMC Hospital, Beijing, 2008–2017.

	*C*. *pneumoniae* IgG Titers ≥1:512 Pos no. (%)	*C*. *pneumoniae* IgM Titers ≥1:16 Pos no. (%)	*C*. *pneumoniae* Acute antibodies Pos no. (%)	*χ*^*2*^-value	*P*-value
AECOPD	15(14.0)	1(0.93)	15(14.0)	**20.43**	0.000*
Pneumonia	145(5.6)	57(2.2)	202(7.8)	**51.87**	**0.000**
≤5 y	2(0.81)	3(1.21)	5(2.0)		
Adult(18y–)	132(6.1)	40(1.9)	177(8.2)	**41.77**	**0.000**
Acute respiratory tract infection	48(10.4)	17(3.7)	57(12.3)	**60.91**	**0.000**
FUO	122(3.8)	38(1.2)	153(4.8)	**0.77**	**0.381**

AECOPD, acute exacerbation of chronic pulmonary diseases; FUO, fever of unknown origin

*Comparison of incidence of acute infection of *C*. *pneumoniae* between patients with AECOPD and all patients (4.4%, 529/12,050), same patterns used for other comparisons in Table 8.

## Discussion

The seroprevalence of past *C*. *pneumoniae* infection is very low in children under 5 years of age, increases dramatically to 50% in young adults of 20 years of age and reaches around 75% in the elderly population [2, 3]. In Japan in 2002, the seroprevalence was 4% in 6 months to 1 year of age, 5% in 2 to 4 year of age, 57% in 20 to 29 year of age and 70% ≥60 year of age subjects [12]. In our study, as shown in Table 1, a prevalence of greater than 20% higher was observed among the middle- or older- aged subjects when compared with the same age groups above. However, our 96% to 98% prevalence in elderly patients seemed consistent with the reports from south Taiwan in 1993, in which the seroprevalence of anti-*C*. *pneumoniae* IgG was 96.2% among study subjects between 71–80 years old [13]. The reason for this high prevalence might be that *C*. *pneumoniae* is spread through person to person by respiratory droplets, and infection or transmission is associated with close contacts and high population density in the areas. For subjects ≤ 2 years old, the prevalence of anti-*C*. *pneumoniae* IgG antibodies was less than 4% in previous reports [2, 3, 12], but our result was around 60%. Why was our result so different? In our view, most of the 60% of the antibodies in this age group were passive immunoglobulins from mothers’ bodies, and in fact, the level was consistent with the seroprevalence of most mothers between 21 to 30 years old. The second point that supports our explanation was that the 60% of antibody prevalence in subjects ≤ 2 years of age decreased to 42.8% in 3–5 years old subjects, rather than increasing with age, indicating the gradual fading away of these passive immunoglobulins. Third point that backing our explanation is that the prevalence of anti-*C*. *pneumoniae* IgG antibodies was 50–63% in newborn cord blood in the studies from south Taiwan, Japan and South Korea [13–15].

Adult men were found to have considerably higher seroprevalence of anti-*C*. *pneumoniae* than adult women in the previous reports [2, 3, 16]. In our study, the sex distribution of seroprevalence was almost equal between men and women before 20 years of age, however, sex differences were observed to be significant in patients after 20 years of age as shown in Table 1. In addition, a study from Finland indicated that GMTs of anti-*C*. *pneumoniae* IgG antibodies, as an indicator of antibody concentration, increased with age and were significantly higher in elderly Finnish men than women [17]. As presented in Table 2 in our study, although GMTs were found to be significantly higher among all women than among all women, sex differences of GMTs actually began to be significant in patients after 40 years of age and the gap enlarged as age increased further. Now the question arises as to what is the meaning of this sex difference in the elderly in both prevalence and concentration of anti-*C*. *pneumoniae* IgG antibodies? It was once speculated by Miyashita et al [12] that “*Chlamydia pneumoniae* infection might contribute to the sex specific difference in the incidence of cardiovascular diseases”, and these debates have persisted for more than two decades [18–23].

Tables 3, 4 and 5 summarize the two peaks of acute infection of *C*. *pneumoniae*. The first peak is primary infection of *C*. *pneumoniae* beginning at 6 years of age in patients with accompanying high frequency of anti-*C*. *pneumoniae* IgM antibodies. In the Chinese schooling system, first grade primary school pupils must be 6 full years old, so it was not difficult to understand that these cases of *C*. *pneumoniae* infection was due to the close contact in school. However, a question remains to be answered, for most children between 3 to 6 years old in Beijing, and most likely in other areas of China, are usually sent by their parents to kindergartens, where the size and crowd situation are similar to the primary schools although there are fewer courses. Why are these children of 3 to 6 years of age are not massively infected with *C*. *pneumoniae*? The second peak of acute infection of *C*. *pneumoniae* in our study appeared in patients who were 60 years old or elderly with an accompanying low frequency of anti-*C*. *pneumoniae* IgM antibodies. This second peak of acute infection was consistent with that found in other studies [13, 17], and the reason for this could be re-infection or chronic infection, to which men seemed more susceptible than women.

*C*. *pneumoniae* infections have been found to occur in all years with wide variation in incidence [3]. As shown in Table 6, the periodicity of the infections was also observed across the decade of our study, yearly variation seemed irregular, and statistical difference were found in some years when compared with the average incidence for the 10 years. *C*. *pneumoniae* infection has been found to have a higher incidence in winter [24, 25], while another study showed that atypical microbes including *C*. *pneumoniae* in community-acquired pneumonia (CAP) were associated with non-respiratory season [26]. In the present study, no statistical variation among the 4 seasons was found although the incidence in winter season seemed to be lower than that in the other 3 seasons.

The average prevalence of acute infection of *C*. *pneumoniae* was found to be 2.9% among children with CAP, who were younger than 5 years old in mainland China in a systematic review [27]. Our study was retrospective, as most patients could not be distinguished to have CAP or to have other forms of pneumonias on doctors’ laboratory application sheets, so we defined CAP and other pneumonias with the general name of pneumonia, and 2.0% (5/248) of patients ≤5 years old with pneumonia were found to have acute antibodies against *C*. *pneumoniae*. For adult patients, Liu et al, [28] reported that *C*. *pneumoniae* was causative agent in 6.6% of adult Chinese patients with CAP in the urban areas. In our study, 8.2% (177/2161) of adult patients with pneumonia were observed to have acute antibodies against *C*. *pneumoniae*. However, there was a difference in methodology for testing of anti-*C*. *pneumoniae*, in which both anti-*C*. *pneumoniae* IgG and IgM antibodies were tested in our study and only anti-*C*. *pneumoniae* IgG antibodies were tested in the study of Liu et al. In our study, there were 19 serum specimens with anti-*C*. *pneumoniae* IgM antibodies but their titers of anti-*C*. *pneumoniae* IgG antibodies were less than 1:512 in the adult patients with pneumonia. If these 19 specimens had not been tested for anti-*C*. *pneumoniae* IgM antibody, the incidence of acute infection of *C*. *pneumoniae* in adult patients with pneumonia would have dropped to 7.3% (158/2161), which could be similar to the 6.6% in the report of Liu et al.

Seropositivity of *C*. *pneumoniae* was an independent risk factor for COPD in a general population in Japan [29]. *C*. *pneumoniae* was associated with a higher rate of exacerbations of COPD and chronic bronchitis [30, 31]. The association was also observed between AECOPD and acute infection of *C*. *pneumoniae* in our study. Moreover, the rate of serological evidence of *C*. *pneumonia*e infection was 10.5% in children with acute respiratory infections in Peru [32] and 12% in children with lower respiratory tract infections in India [33]. In our study, 12.3% (57/462) of patients with acute respiratory tract infection were found to have acute antibodies against *C*. *pneumoniae*. It is presumed that chronic colonization [29] with *C pneumoniae* in the respiratory tract may play a role at initial stage, and followed by other pathogens, or mixed infections, which were reported to be common in previous studies [31, 34, 35]. Many patients in our study might have been tested for other commonly occurring respiratory pathogens. Unfortunately we did not have these data, so patients in our study might be infected with other respiratory pathogens, regardless of presence or absence of acute anti-*C*. *pneumoniae* antibodies.

Based on our experience during the study, we suggest it is advisable that both anti-*C*. *pneumoniae* IgG and IgM antibodies should be tested for serological diagnosis of acute infection of *C*. *pneumoniae*. If anti-*C*. *pneumoniae* IgG antibody is tested alone, the incidence of acute infection of *C*. *pneumoniae* will be underestimated owing to the by missing of specimens with anti-*C*. *pneumoniae* IgM antibodies but with IgG antibodies that do not reach the criteria for acute infection, as in the report from Liu et al. On the other hand, if anti-*C*. *pneumoniae* IgM antibody is tested alone, the incidence will be dramatically underestimated due to the exclusion of specimens without anti-*C*. *pneumoniae* IgM antibodies but with IgG antibodies that do meet the criteria for acute infection. In present study, there were only 141 (1.2%) specimens with anti-*C*. *pneumoniae* IgM antibodies, 402 (3.3%) were detected to have anti-*C*. *pneumoniae* IgG with titers equal to or greater than 1:512, so if only anti-*C*. *pneumoniae* IgM antibody had been tested, at least 2/3 of specimens from patients with acute infection would have been missed, and it has occurred in previous studies [36, 37].

## Supporting information

S1 File Excel database of anti-*C*. *pneumoniae* antibodies between 2008 and 2017. (XLSX)
